# Biomimetic nanoparticles blocking autophagy for enhanced chemotherapy and metastasis inhibition via reversing focal adhesion disassembly

**DOI:** 10.1186/s12951-021-01189-5

**Published:** 2021-12-24

**Authors:** Yesi Shi, Gan Lin, Huili Zheng, Dan Mu, Hu Chen, Zhixiang Lu, Pan He, Yang Zhang, Chao Liu, Zhongning Lin, Gang Liu

**Affiliations:** 1 State Key Laboratory of Molecular Vaccinology and Molecular, Diagnostics & Center for Molecular Imaging and Translational Medicine, School of Public Health, Xiamen University, Xiamen, 361102 China; 2 Department of Anesthesiology, Zhongshan Hospital of Xiamen University, Xiamen, 361004 China; 3 Amoy Hopeful Biotechnology Co., Ltd., Xiamen, 361027 China; 4 State Key Laboratory of Cellular Stress Biology, Innovation Center for Cell Biology, School of Life Sciences, Xiamen University, Xiamen, 361004 China

**Keywords:** Biomimetic, Targeted co-delivery, Autophagy inhibition, Focal adhesions, Metastasis

## Abstract

**Background:**

Autophagy is a conserved catabolic process, which plays an important role in regulating tumor cell motility and degrading protein aggregates. Chemotherapy-induced autophagy may lead to tumor distant metastasis and even chemo-insensitivity in the therapy of hepatocellular carcinoma (HCC). Therefore, a vast majority of HCC cases do not produce a significant response to monotherapy with autophagy inhibitors.

**Results:**

In this work, we developed a biomimetic nanoformulation (TH-NP) co-encapsulating Oxaliplatin (OXA)/hydroxychloroquine (HCQ, an autophagy inhibitor) to execute targeted autophagy inhibition, reduce tumor cell migration and invasion in vitro and attenuate metastasis in vivo. The tumor cell-specific ligand TRAIL was bioengineered to be stably expressed on HUVECs and the resultant membrane vesicles were wrapped on OXA/HCQ-loaded PLGA nanocores. Especially, TH-NPs could significantly improve OXA and HCQ effective concentration by approximately 21 and 13 times in tumor tissues compared to the free mixture of HCQ/OXA. Moreover, the tumor-targeting TH-NPs released HCQ alkalized the acidic lysosomes and inhibited the fusion of autophagosomes and lysosomes, leading to effective blockade of autophagic flux. In short, the system largely improved chemotherapeutic performance of OXA on subcutaneous and orthotopic HCC mice models. Importantly, TH-NPs also exhibited the most effective inhibition of tumor metastasis in orthotopic HCCLM3 models, and in the HepG2, Huh-7 or HCCLM3 metastatic mice models. Finally, we illustrated the enhanced metastasis inhibition was attributed to the blockade or reverse of the autophagy-mediated degradation of focal adhesions (FAs) including E-cadherin and paxillin.

**Conclusions:**

TH-NPs can perform an enhanced chemotherapy and antimetastatic effect, and may represent a promising strategy for HCC therapy in clinics.

**Graphical Abstract:**

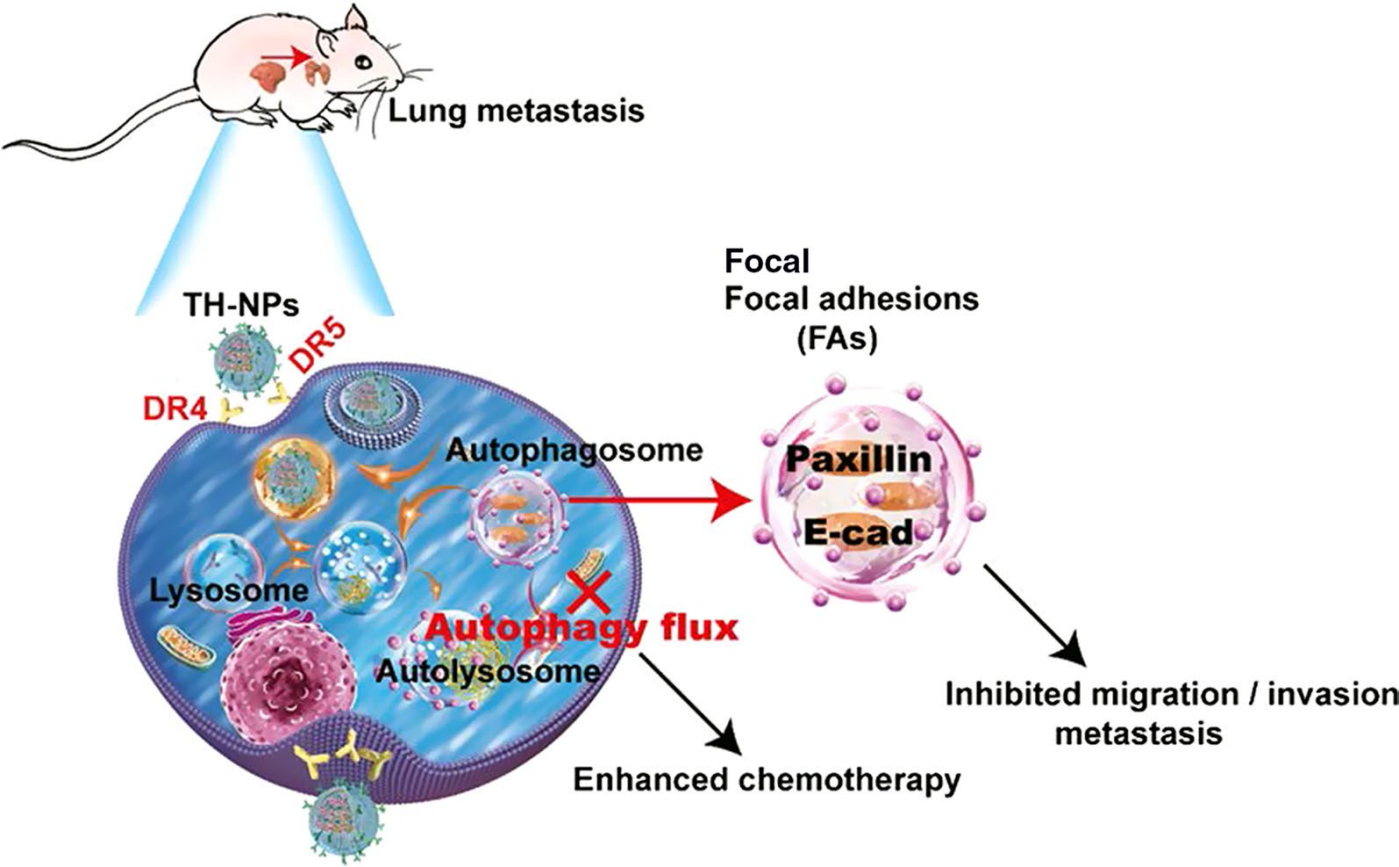

**Supplementary Information:**

The online version contains supplementary material available at 10.1186/s12951-021-01189-5.

## Introduction

Despite the enormous therapeutic potential of chemotherapy, it benefits only a small subset of patients and the response rate of OXA-based chemotherapy in overall patients is unsatisfactory, which limits the application in clinical practice [1–3]. Chemotherapy-triggered protective autophagy plays a crucial role in producing negative chemotherapeutic response rates on cancer patients [4, 5]. Even worse, primary cancer can change to a more aggressive phenotype and accelerate distant metastasis after several courses of chemotherapy [6–8]. Mounting evidences have illustrated the roles of autophagy in regulating the chemosensitivity and metastatic ability of HCC cells [9–11]. The triggered protective autophagy is the process, in which autophagosomes fused with lysosomes to form autolysosomes [12, 13], subsequently degrades protein aggregates or damaged organelles, and is activated during chemotherapy to generate metabolites and energy to meet the metabolic demands of cancer cells [14–16]. Due to the facts, cancer cells can escape from the chemotherapy’s cytotoxicity, which finally facilitates the insensitivity of chemotherapeutics to tumor cells [17, 18].

More seriously, chemotherapy-triggered protective autophagy can decrease the cancer cell adhesions [19, 20], induce tumor cells EMT process [21], and promote distant metastasis or recurrence [22, 23]. Focal adhesions (FAs) play an important role in tumor cell motility, and are reported to aggregate in autophagy-deficient cells [20]. Thus, pharmaceutic manipulation of autophagy may offer a promising strategy for chemosensitization, FAs degradation turnover and metastasis inhibition, considering that the autophagic flux blockade can disrupt the cancer cells' metabolism process and weaken the cancer cells' adaptability [24–26]. Hydroxychloroquine (HCQ) or its derivates have been approved as chemosensitizers for augmenting the therapeutic efficacy of co-administered chemotherapies in clinical trials (NCT03037437 and NCT01897116), clinically reliable autophagic flux blocking agents so far [9, 10]. HCQ, as a weak base, can alkalize and raise the pH value of lysosomes, leading to the lysosomes’ dysfunctions, inhibition of lysosomes fusion with autophagosome and blockade of autophagic flux [27, 28]. In clinics, using HCQ as a monotherapy strategy offers a circumscribed effect to block autophagic flux [29]. However, when HCQ is used as a chemosensitizer and combined with other antineoplastic drugs, it profoundly improves chemosensitivity, reduces drug dosage, and enhances anticancer efficacy [30, 31]. However, the detailed mechanism underlying chemotherapy-triggered protective autophagy, FAs disassembly [32–34] and tumor metastasis is not clearly illustrated.

Oxaliplatin (OXA), a third-generation platinum drug, is used as a combined therapeutic technique for advanced HCC patients [4, 35]. However, its monotherapy has a limited efficacy in clinics, and its induced protective autophagy is responsible for the attenuated chemosensitivity to cancer patients [5, 36, 37]. Inspired by the fact, Food and Drug Administration approved the co-delivery therapy (daunorubicin/cytarabine) for acute myeloid leukemia in 2017, many strategies for co-delivering OXA are developed. Especially, the co-delivery of HCQ/OXA is one of the most potential systems, in which HCQ suppress OXA-triggered protective autophagy, undermine cancer cell energy metabolic homeostasis, and promote chemosensitivity [37]. However, due to poor pharmacokinetics, restricted tumor accumulation and non-specific toxicities to normal organs or tissues, HCQ utility in enhancing the efficacy of OXA-based chemotherapy has been hindered [27, 29, 38]. Lack of tumor targeting remains a major challenge in anti-cancer drug delivery, which severely decreases the probability of drug assimilation by cancer cells [39–41]. More seriously, HCQ monotherapy with a high dosage can suppress the human immune response and weaken the antitumor effect, compared to the effect of autophagic flux blockage [29, 42]. Hence, an urgent need is required to develop a novel co-delivery nanocarrier to address the aforementioned obstacles. Following are the important characterizations of the nanocarrier: (1) simultaneously loading hydrophilic and hydrophobic drugs, (2) endocytosing by cancer cells via the lysosomal pathways and releasing the drug sustainably, (3) accumulating in the tumor sites through active targeting while sparing on normal tissues.

Herein, we develop the biomimetic and tumor targeting co-delivery nanocarrier for autophagy inhibition-based OXA-combined therapy to enhance chemosensitivity and reduce cancer metastasis in HCC. The amphiphilic polymer polylactic-co-glycolic acid (PLGA) is used to synthesize the inner core owing to its favorable biodegradability, biocompatibility, controlled and sustained-release properties [39]. In addition, hydrophilic HCQ and hydrophobic OXA are co-encapsulated into the nanocores. Furthermore, tumor-specific targeting protein, tumor necrosis factor-related apoptosis-inducing ligand (TRAIL), can induce apoptosis of cancer cells upon binding death receptor 4/5 (DR4/5) without causing toxicity on normal cells or tissues.

Therefore, TRAIL is genetically displayed on the surface membrane of the human umbilical vein endothelial cell (HUVEC) due to its potential and preferred tumor tropism [43, 44]. The TRAIL overexpressing HUVEC membrane vesicles (TH-V) is extracted and coated onto the tailor-designed drug-loaded PLGA NP (TH-NP), according to the outcomes of our previous studies [43, 45]. The outer cell vesicles of TH-NPs tactfully prolong their circulating time in vivo*,* inhibiting the engulfment by macrophage because of the CD47 and other important functional membrane proteins [40]. Due to the existence of TRAIL on the outer shell, the TH-NPs actively accumulate into the tumor site through TRAIL binding to the DR4/5 on HCC cells, reducing the non-targeting biodistribution of the HCQ and OXA drugs. Afterward, the TH-NPs dissociate in the low-pH microenvironment. The released HCQ alkalizes and damages the lysosomes, thereby promoting HCQ/OXA escape from lysosomes. After the lysosomes damage, autophagic flux inhibition facilitates the chemosensitivity of the coloaded OXA against HCC cells. Besides, the autophagic flux blockade further limits the FAs includes E-cadherin and paxillin disassembly or degradation, thus inhibiting tumor metastasis. In addition, the TH-NPs can bind to the spread cancer cells and reduce metastasis in HCCLM3, HepG2 and Huh-7 metastatic mouse models. Collectively, the developed co-delivery nanoparticle TH-NP can efficiently suppress tumor growth and tumor metastasis in vitro and vivo, which represents a promising strategy for HCC therapy in clinics.

## Results and discussion

### Development of targeted OXA/HCQ co-delivery nanocarrier.

To validate whether HCQ can produce a synergistic effect with OXA on HCC cells, a crystal violet staining assay was performed in HepG2, Huh-7, and HCCLM3 cells. After incubation and washing, the results showed that HCQ/OXA mixture treatment can inhibit the colony formation of HCC cells more efficiently than free HCQ or OXA monotherapy (Additional file 1: Figure S1A). To further evaluate whether the synergistic effect was associated with autophagy, the HCCLM3 GFP-LC3 overexpressing cells were co-cultured with the indicated drugs. The results showed that GFP-LC3 puncta aggregated more in the cells treated with the HCQ/OXA than other treatments, indicating HCQ blocked OXA-triggered protective autophagy and enhanced OXA chemosensitivity, confirming by the increased expression of cleaved PARP (Additional file 1: Figure S1B–D). Moreover, the HepG2 and Huh-7 cells also exhibited the same effects (Additional file 1: Figure S1E). However, in some clinical trials (NCT01206530 and NCT010063690), the free HCQ/OXA therapy failed to achieve desired clinical outcomes, due to the lack of effective nanocarriers to precisely deliver autophagy inhibitors and chemotherapeutics to cancer tissues while sparing on normal tissues.

**Fig. 1:**
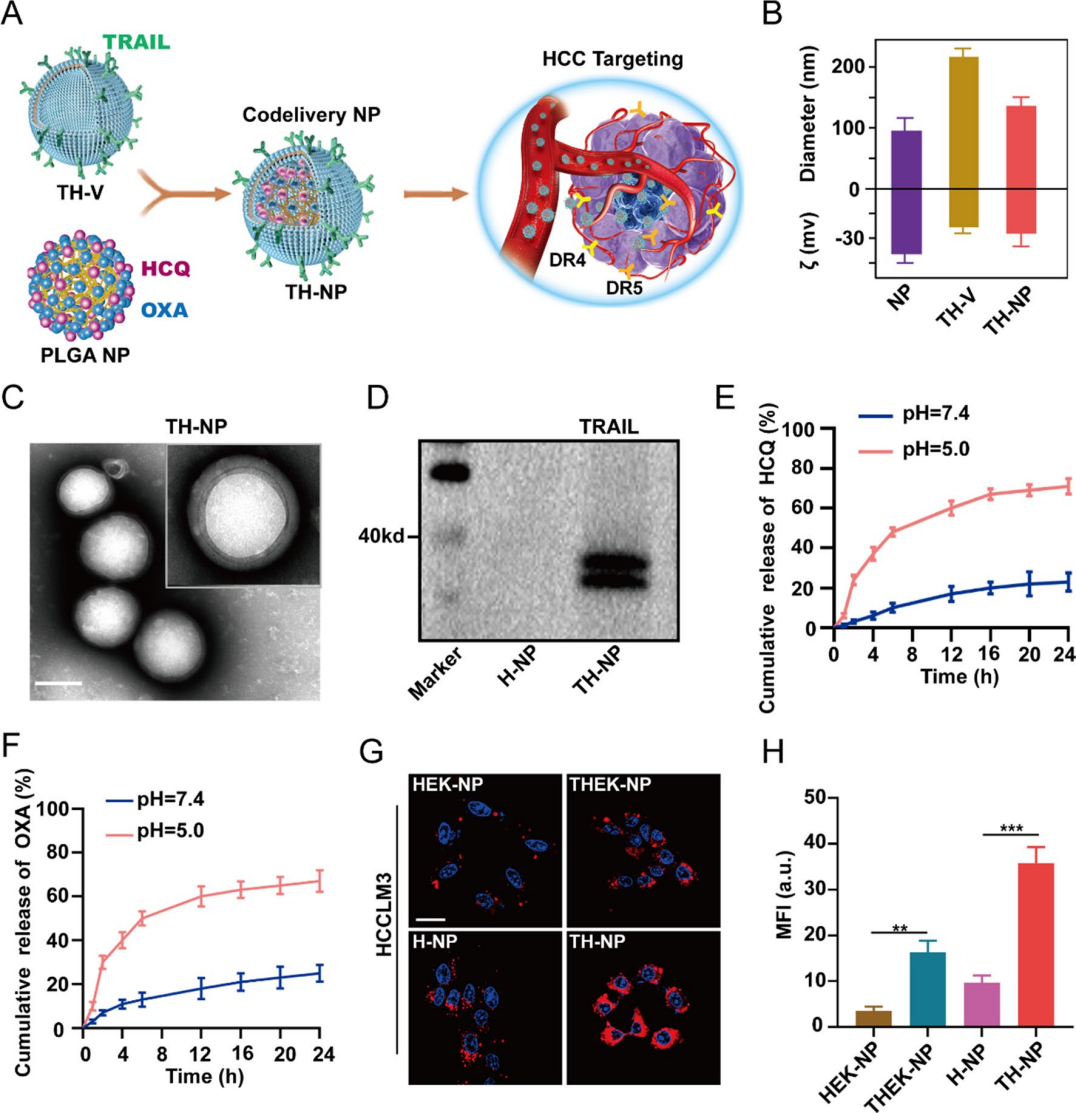
Preparation of tumor-targeting nanoformulation TH-NP for the co-delivery of OXA and HCQ. **A** Schemes showing the fabrication of TH-NP and usage as an HCC targeted co-delivery system, which was synthesized through TRAIL expressing HUVECs membrane coating on PLGA NPs loading OXA and HCQ. **B** DLS measurements (hydrodynamic size and zeta potential ζ) of TH-NP. **C** TEM of the targeted co-delivery NPs TH-NPs, showing the intact membrane coating onto PLGA NPs and the uniform size distribution. Scale bar:100 nm. **D** Western blotting results showing TRAIL protein was integrally preserved on the outer membrane of TH-NPs without damaging its activity. **E** Release profiles of HCQ from the TH-NPs. **F** Release profiles of OXA from the TH-NPs. **G** Confocal microscopy displayed the tumor targeting of Dil-conjugated various membrane coated NPs in the HCCLM3 cells. TH-NPs showed the most potent HCC targeting compared to H-NPs, HEK-NPS or THEK-NPs. (Dil, red; Hoechst, blue). Scale bar: 20 μm. **H** Comparison of relative mean fluorescence intensity (MFI) of different nanoparticles of HCC targeting. Data were displayed as the mean ± SD. *P < 0.05; **P < 0.01; ***P < 0.001

Herein, we developed tumor-targeting and biomimetic drugs co-delivery nanoparticles, comprising of cell-derived membrane vesicles, tumor targeting, and drug co-loading PLGA NPs to achieve targeting co-delivery of chemotherapeutic and HCQ to the cancer tissues. Gene engineering was used to construct TRAIL overexpressing HUVECs. Then, the cell membrane vesicles were extracted and wrapped onto the synthesized HCQ/OXA co-capsulated inner cores as illustrated in Fig. 1A. The immunofluorescence images showed that TRAIL-GFP fusion protein was successfully displayed on the surface (Additional file 1: Figure S2A), which presented the subsequent TH-NPs with HCC tumors targeting through TRAIL-DR4/5 interactions (Additional file 1: Figure S2B). Respectively, the mean hydrodynamic size and zeta potential of NPs, TRAIL expressing HUVECs vesicles (TH-Vs), and TRAIL expressing HUVECs vesicles coated NPs (TH-NPs) were measured by the dynamic light scattering (DLS). The results were NPs (85 nm, − 35 mV), TH-Vs (210 nm, − 16 mV), and TH-NPs (120 nm, − 20 mV) (Fig. 1B). The transmission/scanning electron microscopy (TEM/SEM) were also used for the physical characterization (Fig. 1C and Additional file 1: Figure S3A). Importantly, the TH-NPs size increased by approximately 30 nm compared to that of bare NPs, indicating that the TH-Vs were successfully coated onto the NPs. Moreover, the TH-NPs negative charge may avoid being cleared by the reticuloendothelial system (RES), thereby prolonging the circulation time in vivo [41, 42]. To investigate its stability, TH-NPs underwent repeated freezing and thawing three times, and the results showed that the size of TH-NPs remained the same, indicating long-term storage for future application (Additional file 1: Figure S3B). Importantly, active TRAIL protein (Fig. 1D) and other functional molecules such as CD44, CD47, and ICAM-1 (Additional file 1: Figure S3C) were detected on the TH-NPs surface through western blotting, which indicated the properties of cell membrane inheritance without damaging the membrane vesicles integrity. Respectively, the encapsulation efficiency (EE) of HCQ and OXA in TH-NPs were 81.5% and 88.9% (Additional file 1: Figure S3D). The HCQ and OXA drug loading efficiency (DLE) in the NPs was separated by approximately 11.5% and 5.6%. The outer cell membrane vesicles and PLGA cores present TH-NPs, which is the property of controlled drug release on-demand. The cumulative release results showed that HCQ and OXA in TH-NPs released approximately 76.3 and 70.5% at pH = 5.0, about 5.01 and 3.56 times at pH = 7.4 (Fig. 1E, F). The results indicated that TH-NPs could release more drugs in the acidic tumor microenvironment and lower drugs in the adjacent normal tissues.

As illustrated previously, TRAIL can improve tumor targeting. Here, Dil labeled H-NP and TH-NP were co-incubated with HCCLM3 cells. HEK-293T cell membrane coated NP (HEK-NP) and TRAIL-expressing HEK-293T cell membrane coated NP (THEK-NP) were tested as controls because they had similar particulate structures as TH-NP, but HEK-293T cells are less plastic in promoting or enhancing tumor targeting compared to HUVECs. After incubation and washing, significant fluorescence was observed on TH-NP treated cells, but not H-NP. THEK-NP treated cells also showed a higher fluorescence intensity than HEK-NP, but not more than that of TH-NP (Fig. 1G, H). Moreover, the similar effects were also found on HepG2 and Huh-7 cells (Additional file 1: Figure S4A). To further confirm the tumor-targeting efficiency of TH-NPs, photoacoustic imaging (PAI) was also used for assay. Free ICG, ICG-labeled NP, HEK-NP, THEK-NP, H-NP, and TH-NP were incubated separately with the high-metastatic HCCLM3 cells. The low-metastatic HepG2, Huh-7 cells, and human normal liver cell LO2 served as a control. After 3 h incubation, tumor-specific targeting was evaluated through PAI signals (Additional file 1: Figure S4B and C). Comparatively, in the three kinds of liver cancer cells, the bare NP treated group exhibited stronger PAI signals than that of free ICG. However, it was substantially lower than the other cell membrane coated nanoparticles treated group. The results showed that bioinspired membranes can facilitate tumor targeting and endow them with the properties of source cells. TH-NP can selectively bind to the three HCC cells, as these cells exhibited the strongest PAI signal. THEK-NP treated group also showed enhanced PAI signals, higher than that of the HEK-NP or H-NP, but weaker than TH-NP. In short, these results demonstrated the ability of TH-NPs to target tumor cells conferred by their TRAIL-expressing membrane coating. The binding was probably attributed to specific interactions between TRAIL on the HUVEC membrane and the DR4/5 overexpressed on HCC cells.

### TH-NPs show a targeted autophagy inhibition

**Fig. 2:**
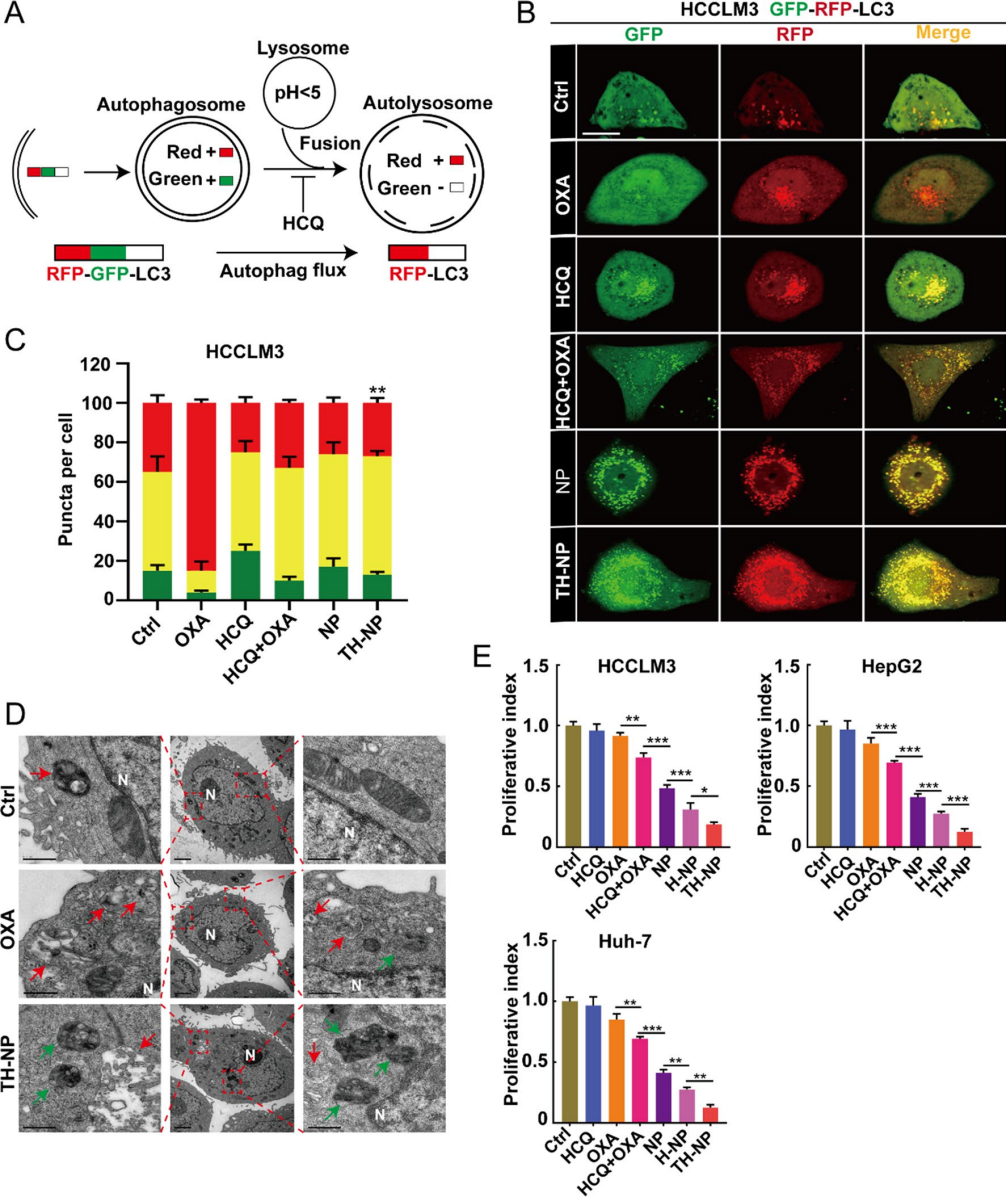
TH-NPs demonstrated targeted inhibition of the protective autophagy and significantly enhanced chemotherapy in vitro. **A** GFP-RFP-LC3 dual reporter assay to analyze the blockade of autophagy flux. **B** The abundance of RFP-LC3 puncta was significantly enhanced over GFP puncta in OXA-treated cells, illustrating OXA triggered protective autophagy indicated by the accumulation of autolysosomes. HCQ can inhibit the autophagy flux or autophagosome–lysosome fusion (Free OXA = 20 μM, HCQ = 10 μM, the equivalent concentration of OXA or HCQ in NPs or TH-NPs). Scale bar: 10 μm. **C** Quantification analysis of the autophagy flux of HCCLM3 after the indicated treatments. Eight random fields (n = 8) were used for quantificationally analyzing the intracellular level of autophagosomes (GFP and RFP puncta) and autolysosomes (RFP-only puncta). **D** Representative TEM images of intracellular level of autophagic vacuoles in HCCLM3 cells (N, nucleus; red arrow, autolysosomes; green arrow, autophagosomes). **E** Quantification of the proliferation index of HCCLM3, HepG2 and Huh-7 cells according to the crystal violet staining after the indicted treatments. Data were displayed as the mean ± SD. *P < 0.05; **P < 0.01; ***P < 0.001

To investigate the interactions between the release performance of TH-NPs, OXA-triggered protective autophagy, and HCQ-mediated blockade, GFP-RFP-LC3 overexpressed HCC cell lines were constructed. GFP is sensitive to acidity and quenched in the low-pH lysosomes, while RFP can keep stable in lysosomes or autolysosomes (Fig. 2A). Due to the fusion protein, LC3-engineered HCC cells synchronously expresses RFP and GFP fluorescence, and hence we can observe the yellow puncta in the autophagosomes. When the autophagic flux was induced, the autophagy marker protein LC3 aggregated in the autophagophores membrane to form autophagosomes. Then, the autophagosomes fused with acidic lysosomes to form autolysosomes, GFP was degraded and only RFP fluorescence can be observed. In short, the changes of yellow puncta (autophagosomes), and the RFP puncta (autolysosomes) reflect that autophagic flux was unobstructed or blocked.

Respectively, more RFP puncta were accumulated in OXA-treated cells compared to control, indicating a protective autophagic flux, and explained the subsequent drug resistance or insensitivity (Fig. 2B and Additional file 1: Figure S5A). In HCQ-treated group, the yellow puncta distinctly aggregated and RFP puncta decreased, revealing the blockade of autophagic flux and remain on the autophagosome stage. By contrast, bare NPs largely induced yellow puncta and RFP puncta, while decreased the RFP ratio in total puncta, illustrating an enhanced autophagic level compared to that of HCQ/OXA (Fig. 2C and Additional file 1: Figure S5B). Obviously, the autophagic level of TH-NPs-treated cells was promoted compared to that of HCQ/OXA, bare NPs, or H-NPs. However, the ratio of RFP puncta or autolysosomes in total puncta was reduced, meaning the autophagy flux was inhibited. Overall, the results demonstrated that TH-NPs could improve the autophagic level, but largely limit autophagic flux, which was superior to other controls or the non-targeting NPs or low-targeting H-NPs. In addition, plenty of autolysosomes (red arrow) and a few autophagosomes (green arrow) were observed in the OXA-treated HCCLM3 cells through TEM images of cells (Fig. 2D and Additional file 1: Figure S5C and D), confirming OXA-based chemotherapy could trigger the protective autophagy. Comparatively, TH-NPs-treated HCCLM3 cells had a fewer amounts of autolysosomes and increased autophagosomes. The autophagosomes/autolysosomes ratio in the group was higher than other groups, illustrating that the protective autophagic flux was inhibited during the fusion process of lysosomes and autophagosomes.

### TH-NPs enhance chemotherapy in vitro

To investigate whether TH-NPs enhanced the chemotherapeutic efficacy, the immunofluorescence (IF) of the tumor cell proliferative marker Ki67 was performed. The downregulated Ki67 expression illustrated that the TH-NPs significantly enhanced cancer proliferation inhibition (Additional file 1: Figure S6A). Crystal violet staining was also performed on HCCLM3 cells to assay the anti-colony formation effect, and the results demonstrated that TH-NPs displayed the optimal anti-colony formation (Additional file 1: Figure S6B). The quantitative analysis of the proliferation index after the treatments with the indicated drugs also confirmed the conclusion on HepG2 and Huh-7 cells. (Fig. 2E). Moreover, the increased apoptosis-related protein cleaved PARP expression also confirmed that the TH-NPs could enhance OXA cytotoxicity or chemosensitivity to cancer cells (Additional file 1: Figure S6C). Collectively, through targeting inhibition of OXA-induced protective autophagy flux, TH-NPs demonstrated the most effective anti-proliferative effect on HCC cells compared to other groups. The enhanced synergistic antitumor effect of TH-NPs in vitro was probably attributed to the TRAIL-DR4/5 axis-based tumor-targeting design.

### TH-NPs limit focal adhesions disassembly via inhibiting autophagy

**Fig. 3:**
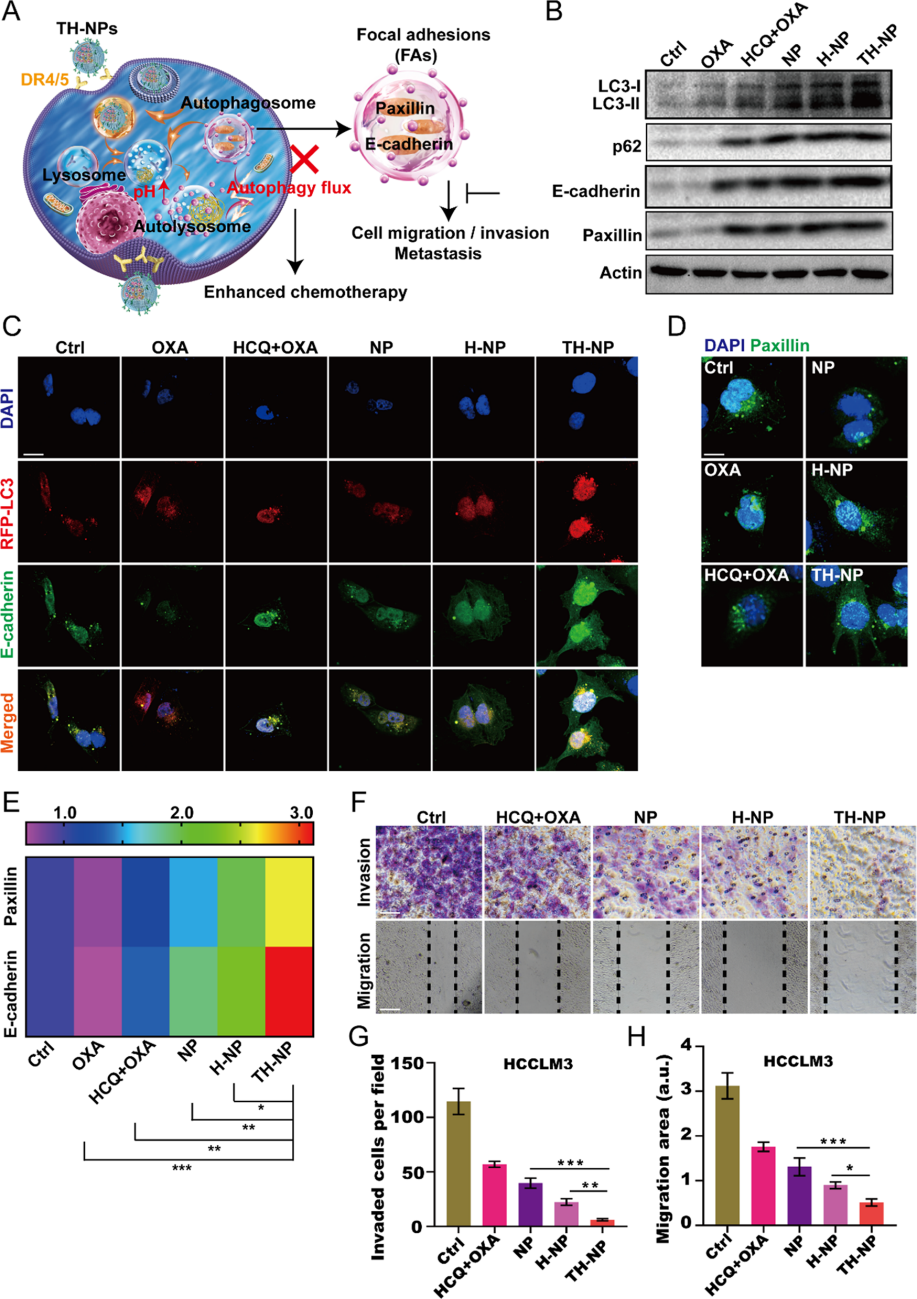
TH-NPs limited tumor invasion and migration by blocking autophagy flux in vitro.** A** Schematic diagram showing blockade of chemotherapy-induced autophagic flux can limit tumor invasion and migration by decreasing FAs disassembly. **B** Western blotting analysis of the autophagy marker proteins LC3 and p62, and its downstream pathway proteins FAs including E-cadherin and paxillin at the cell level. **C** IF images demonstrating OXA-triggered protective autophagy degrades E-cadherin and **D** paxillin, TH-NPs treatment would reverse or increase E-cadherin and paxillin expression. Scale bar: 20 μm. **E** Quantitative analysis of the E-cadherin and paxillin of the IF results. **F** Images of the TH-NPs on invasion and migration of HCCLM3 cells in vitro. **G** The quantitative analysis of invaded cells and **H** migration area of TH-NPs on HCCLM3 cells. Data were displayed as the mean ± SD. *P < 0.05; **P < 0.01; ***P < 0.001

To understand the mechanism between the chemotherapy-induced protective autophagy and metastasis, we explored the potential signaling pathways (Fig. 3A). We firstly detected the autophagic flux and FAs expression by western blotting, and the results showed OXA treatment induced protective autophagic flux, indicated by the increased LC3-II and decreased p62 (Fig. 3B and Additional file 1: Figure S7A). By contrast, free drug pair OXA/HCQ limited the autophagic flux to an extent, but weaker than that of NPs, H-NPs, or TH-NPs. Especially, TH-NPs had the strongest ability to limit autophagic flux, presumably due to the specific interaction TRAIL-DR4/5 axis. Interestingly, the tumor metastatic marker proteins E-cadherin and paxillin reduced after OXA treatment, while TH-NPs treatment could largely reverse FAs reduce. The treatments of HCQ/OXA, NPs and H-NPs could inhibit the FAs degradation to an extent, but weaker than TH-NPs. In short, the more inhibition of protective autophagy flux, probably leading to the less FAs degradation. Moreover, IF experiments were performed to further detect E-cadherin and paxillin on RFP-LC3 HCCLM3 cells. The results illustrated that monotherapy of OXA induced protective autophagy, and subsequently degraded E-cadherin (Fig. 3C). When combined with autophagy inhibitor HCQ, it can delay the FAs degradation. However, when using the rationally designed TH-NPs to treat HCCLM3 cells, the degradation or disassembly was largely reversed or even upregulated. Paxillin after the indicated treatments also showed similar changes (Fig. 3D, E). These data supported that the induced autophagy could degrade FAs while blockade of autophagy flux may reverse the degradation. Together, TH-NPs can block the protective autophagy-mediated FAs disassembly or degradation.

### TH-NPs inhibit tumor invasion and migration in vitro

To investigate whether the FAs degradation was associated with tumor metastasis, transwell invasion and wound-healing assay were performed on low-metastatic HepG2 and Huh-7 cells, as well as on high-metastatic HCCLM3 cells. For the invasion assay, BD matrigel was wrapped onto the well upper chamber to mimic tumor extracellular matrix for cancer cell invasion. Then, the number of cells that crossed the polycarbonate membrane was calculated to reflect the invasive ability. The number of invasive cells per view in H-NPs or NPs-treated group was counted and significantly less than that of control or free HCQ/OXA. It demonstrated that NPs or H-NPs could suppress cancer cell invasion to an extent (Fig. 3F and Additional file 1: Figure S7B). Comparatively, TH-NPs-treated group had the least number of invasive cells (Fig. 3G and Additional file 1: Figure S7C), indicating that TH-NPs had the optimal anti-invasion ability. Similar to the invasion assay, the TH-NPs largely inhibited cancer cell migration, validated by the less migration area compared to other groups (Fig. 3H and Additional file 1: Figure S7D). Collectively, the TH-NPs can target HCC cells to release HCQ to inhibit the fusion between autophagosomes and lysosomes, and thus suppressed protective autophagic flux. Due to the blockade of autophagy flux, TH-NPs further limited the metastatic marker proteins FAs degradation and finally reduced tumor invasion and migration.

### Enhanced antitumor efficacy of TH-NPs in HCC tumors

**Fig. 4:**
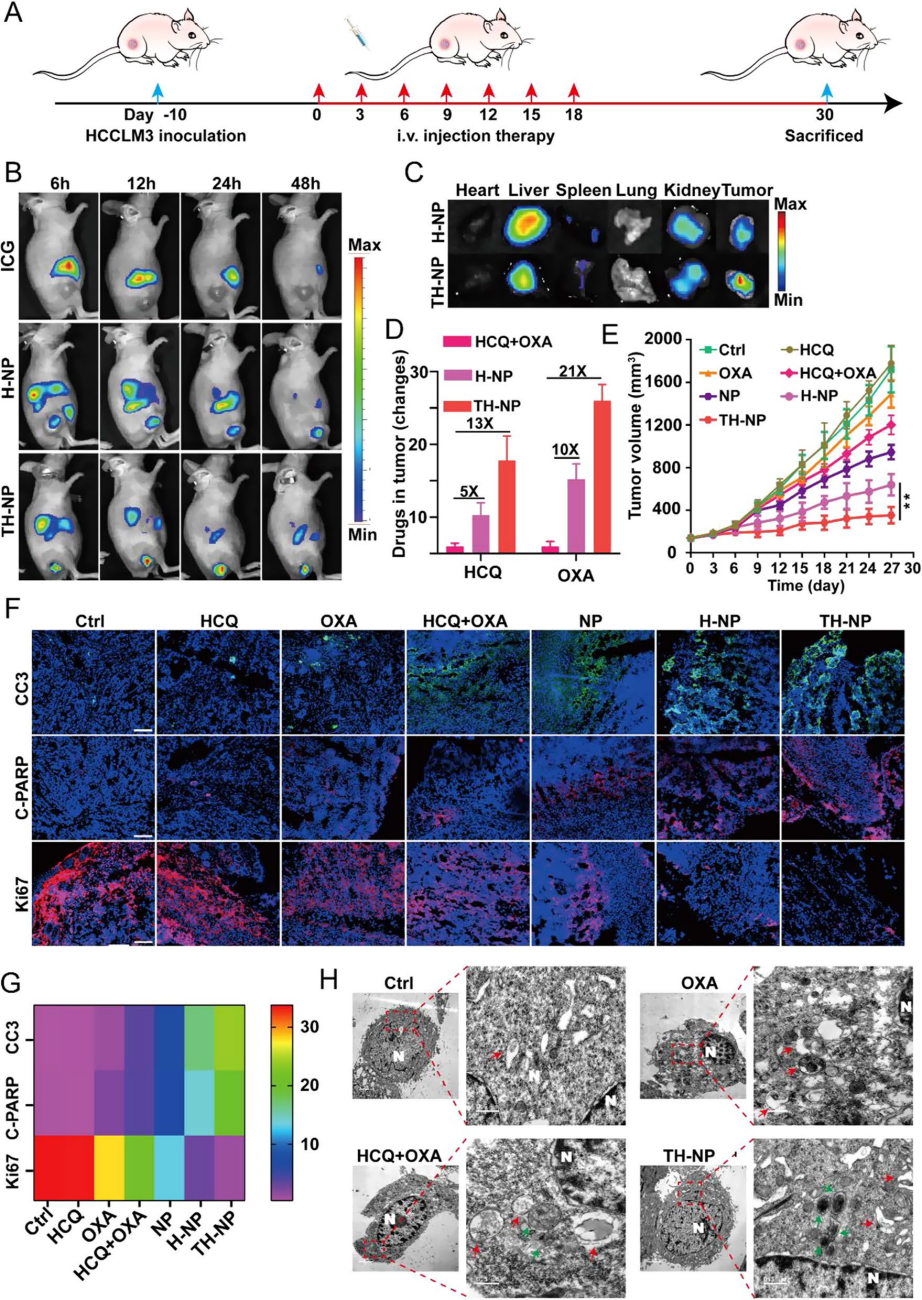
TH-NPs enhanced chemotherapy on HCCLM3 subcutaneous tumor models via autophagy inhibition. **A** Schedule of the antitumor treatment on subcutaneous HCCLM3 xenograft models. Tumor bearing mice (n = 8) with the volume about 150 mm^3^ received i.v. injection of the indicated drugs every three days for seven times. The injection dosages at equivalent dosage of OXA (10 mg/kg) or HCQ (20 mg/kg) in free drugs or NPs, H-NPs or TH-NPs. Mice treated with saline was served as control. Tumor volumes were measured for 27 days and sacrificed for the next analysis on day 30. **B** In vivo IVIS imaging was performed to monitor the biodistribution of TH-NPs on subcutaneous tumor-bearing nude mice (n = 3). Mice were intravenously injected with free ICG (1.5 mg/kg), or equivalent ICG-labeled H-NPs or TH-NPs and the FL images were obtained at 6, 12, 24 and 48 h post injection. **C** Ex vivo IVIS imaging was used to examine the ICG FL signals of major organs and tumor to further study the biodistribution of the nanoparticles, after the mice were sacrificed at 48 h post injection. **D** OXA and HCQ content in the tumor tissues. On day 18, three mice in OXA/HCQ combination, H-NPs or TH-NPs group were sacrificed at 24 h post *i.v.* injection to compare the content of OXA and HCQ. **E** Growth curves of tumors after different treatments. TH-NPs had the most significant tumor growth limitation compared to other groups. **F** Immunohistochemical frozen sections (IHC-F) of tumor tissues of the different groups and **G** its heat map of quantitative analysis. Scale bar: 200 μm. **H** TEM images of the autophagic vacuoles in the tumor tissues of different groups. (N, nucleus; red arrow, autolysosomes; green arrow, autophagosomes). Data were displayed as the mean ± SD. *P < 0.05; **P < 0.01; ***P < 0.001

To examine the therapeutic potential of TH-NPs in vivo, we firstly investigated the anti-tumor efficacy on the nude mice bearing HCCLM3 subcutaneous tumor. The therapeutic regiment was shown in Fig. 4A. Furthermore, to verify the TRAIL-mediated active tumor-targeting efficacy, the time-lapse fluorescence imaging was performed after equivalent free ICG, ICG-labeled H-NPs or TH-NPs were injected into the mice. Since TH-NPs was fabricated using TRAIL-anchored membrane, it facilitated TH-NPs accumulate in the tumor tissues, indicated by the strongest fluorescence than H-NPs or free ICG at the indicated time points (Fig. 4B). Next, Ex vivo fluorescence images of major organs were harvested on 48 h after the injections, and a higher fluorescence in TH-NPs treated tumors was observed, demonstrating the tumor targeting ability of TH-NPs was conferred by TRAIL-anchored cell membrane coating (Fig. 4C). We also quantified the relative fluorescence signals of the major tissues in the two groups and found that the fluorescence intensity of tumors in TH-NP group was approximately 1.8-fold higher than that of H-NP (Additional file 1: Figure S8A). Importantly, the remarkable tumor targeting of TH-NPs was attributed to the outer layer TRAIL protein-DR4/5 on tumor cell axis interactions, which promoted targeting delivery of OXA and HCQ to tumor tissues. To verify the hypothesis, we measured the content of OXA and HCQ in the non-targeting HCQ + OXA, low-targeting H-NPs or TH-NPs group. The results showed that the TH-NPs can significantly improve OXA and HCQ concentration with approximately 21 and 13-fold increment in tumor tissues compared to the free mixture of HCQ/OXA, while H-NPs just improved 10 and fivefold increment (Fig. 4D). It indicated the enhanced chemotherapy of TH-NPs was conferred by the targeted drug delivery.

Respectively, the NPs, H-NPs, or TH-NPs (containing equivalent dosages of OXA: 10 mg/kg and HCQ: 20 mg/kg) were administrated into HCCLM3 tumor-bearing mice once every 3 days for seven times. Free OXA, HCQ or OXA/HCQ mixture treatments served as controls. The tumors in control groups grew very fast, and free HCQ or OXA failed to significantly suppress the tumor growth (Fig. 4E). The HCQ/OXA mixture inhibited tumor progress to an extent, showing that autophagy limitation enhanced OXA-based chemotherapy, but the inhibition was weaker than that of OXA/HCQ loaded NPs or H-NPs. By contrast, after the TRAIL-HUVECs membrane coating, TH-NPs displayed the most potent antitumor effect due to TRAIL-mediated tumor preferential targeting. Furthermore, TH-NPs showed a good biocompatibility and low-toxicity, verified by the little changed body weight (Additional file 1: Figure S8B). Immunohistochemical frozen (IHC-F) were performed to further evaluate the antitumor effect. Consistently, it was observed that the TH-NPs induced the abundance of apoptosis-related proteins including cleaved caspase-3 (CC3) and cleaved PARP (C-PARP) (Fig. 4F, G). In addition, Ki-67 was also detected, and the weakest fluorescence signals were detected in the TH-NPs group compared to other treatments. To examine whether the improved chemotherapy was associated with the enhanced autophagy inhibition, TEM images of cancer tissues were used for analysis. (Fig. 4H and Additional file 1: Figure S8C). Obviously, the increased amounts of autolysosomes showed that OXA treatment triggered protective autophagy, which was responsible for OXA insensitivity. The ratio of the autophagosomes/autolysosomes in the TH-NPs group was significantly higher than that of other groups, indicating a blockade of autophagic flux. Above all, the results confirmed TH-NPs could enhance OXA therapeutic potential by blocking protective autophagic flux while sparing on normal cells. Furthermore, these results also illustrated TH-NPs generated a promising antitumor effect in tumor-bearing mice, indicating the success of pharmaceutical manipulation of autophagy in anticancer applications.

### TH-NPs demonstrate an enhanced antimetastatic activity in vivo

**Fig. 5:**
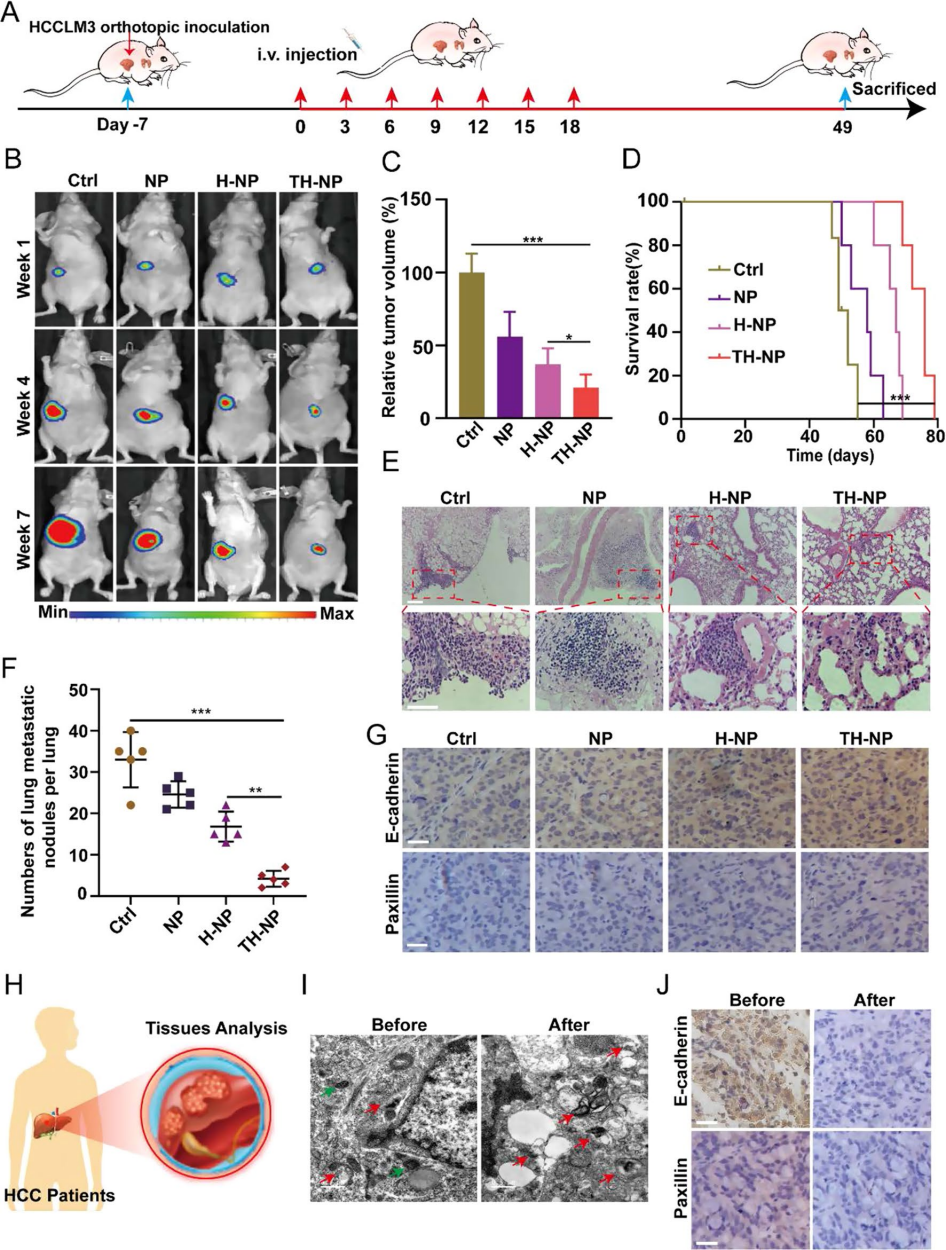
TH-NPs inhibited tumor metastasis by reversing FAs disassembly in orthotopic HCC models. **A** Schedule of the treatments on HCCLM3 orthotopic tumor models. **B** Orthotopic HCCLM3 tumor sites (n = 4) at the indicated week were localized by bioluminescence-based imaging. Mice received i.v. injection of saline, NPs, H-NPs, or TH-NPs at equivalent dosages (OXA: 10 mg/kg; HCQ: 20 mg/kg). **C** The relative tumor volume was quantified by bioluminescence intensity, and TH-NPs treatment showed the highest orthotopic tumor inhibition. **D** Kaplan–Meier survival plot of HCCLM3 orthotopic tumor-bearing mice after *i.v.* injection of the indicated formulations. **E** H&E staining of lung metastatic nodules of HCCLM3 tumor-bearing mice at the end of the experiments, Scale bar: 100 μm. **F** The numbers of lung metastatic nodules in the lungs. **G** IHC staining showing focal adhesions including E-cadherin and Paxillin increase after TH-NPs treatments, which limited tumor distant metastasis. Scale bar: 100 μm. **H** Scheme showing the cancer tissues of HCC patients were obtained based on the following analysis. **I** TEM images of the autophagic vacuoles in the HCC patients before or after chemotherapy. (N, nucleus; red arrow, autolysosomes; green arrow, autophagosomes). Scale bar: 0.5 μm. **J** IHC staining of E-cadherin and paxillin. The patients who received OXA-based chemotherapy (after) had a low expression compared to the patients without chemotherapy (before). Scale bar: 100 μm. Data are displayed as the mean ± SD. *P < 0.05; **P < 0.01; ***P < 0.001

To investigate the cross-reactive antitumor and metastasis inhibiting efficacy of the TH-NPs, the orthotopic HCCLM3 tumor-bearing mice were developed. On day 7, after the inoculation of HCCLM3-Luc cells, the mice with similar tumor volume were chosen and randomly divided into four groups. The mice received *i.v.* injection of saline, bare NPs, H-NPs, or TH-NPs at an equivalent dosage of OXA or HCQ every three days seven times (Fig. 5A). The targeted co-delivery TH-NPs showed an enhanced antitumor efficacy in orthotopic HCCLM3 mice, according to the bioluminescence (Fig. 5B), showing approximately 74% decrease in tumor volume compared to the control, whereas bare NPs (45%) and H-NPs (61%) (Fig. 5C). Moreover, TH-NPs also significantly prolonged the mean survival time compared with other controls (Fig. 5D). For the antimetastatic studies, the H&E staining of the enlarged lungs images showed that the TH-NPs deduced the number of lung metastatic nodules and narrowed the diameter of the nodules (Fig. 5E and Additional file 1: Figure S9A). The quantitative results exhibited that both NPs and H-NPs suppressed cancer metastasis, but weaker than that of the TH-NPs. The average number of lung metastatic nodules in TH-NPs shrank about 25, 17, and 9 per lung than that of the control, NPs, and H-NPs group (Fig. 5F). To further evaluate the broad-spectrum antimetastatic activity of TH-NPs, the HCCLM3, HepG2 and Huh-7metastatic mouse models were established. After 48 h intravenous injection of 2 X 10^6^ HepG2, Huh-7 or HCCLM3 cells into nude mice, the mice were then injected with TH-NPs intravenously once every 3 days for 18 days (Additional file 1: Figure S9B). H&E staining showed fewer lung metastatic nodules of the three HCC metastatic models in TH-NPs treated groups compared with that of control groups (Additional file 1: Figure S9C). The quantitative analysis of lung metastasis nodules of TH-NPs-treated mice also confirmed the conclusion (Additional file 1: Figure S9D). Collectively, the results illustrated the excellent antimetastatic efficacy of TH-NPs, regardless of tumor models.

### Blockade of autophagy leads to the reverse of FAs turnover

As FAs degradation was closely related to cancer metastasis, we hypothesized that the degradation process involved lysosomes or autophagosomes, and pharmaceutical manipulation of autophagy might reverse the degradation. To reveal the mechanism, we analyzed the autophagic level of the tumors after the indicated treatments. From the TEM images, we observed that the tumor in the control group had more autolysosomes and higher lung metastasis nodules. However, the autolysosomes significantly decreased and autophagosomes increased in the tumor of the TH-NPs group (Additional file 1: Figure S10A) with fewer metastatic nodules. When treated with TH-NPs, the FAs including E-cadherin and paxillin significantly increased (Fig. 5G and Additional file 1: Figure S10B), which enhanced tumor cell adhesions and reduced epithelial-mesenchymal transition (EMT) and subsequent cancer metastasis. The NPs or H-NPs treatment also improves the expression of E-cadherin and paxillin, but weaker than that of the TH-NPs. These results demonstrated that TH-NPs could achieve promising anticancer effects and metastasis inhibition in the orthotopic or metastatic mouse models through inhibiting autophagic flux to reverse FAs disassembly.

TH-NPs have achieved enhanced antitumor effects and metastasis inhibition in vitro or in vivo. To verify the mechanism between the chemotherapy-induced protective autophagy and metastasis in clinics, we used IHC to assay the expression of FAs including E-cadherin and paxillin of HCC patients before or after receiving OXA-based chemotherapy (Fig. 5H). The patient’s tumor tissues with chemotherapy showed a high level of autophagy compared with that of patients without chemotherapy (Fig. 5I). Furthermore, at low magnification, the IHC staining showed the reduced expression of E-cadherin and paxillin in the patients who received chemotherapy (Fig. 5J). Collectively, these data indicated that chemotherapy-induced autophagy promoted FAs disassembly and tumor metastasis. This may give us an important hint to pay special attention to the induced autophagy during the chemotherapy. Importantly, TH-NPs can effectively block the autophagy-mediated FAs degradation and thus inhibit tumor metastasis and enhance chemotherapy, may represent a potential strategy for limiting tumor metastasis in clinics.

## Discussions and conclusions

Due to the TRAIL-DR4/5 axis, TRAIL-based nanoformulation was developed for targeted and disease-related delivery of antitumor drugs and autophagy inhibitors, leading to enhanced antitumor efficacy. In the study, we validated an active tumor-targeting nanocarrier co-delivering chemotherapeutic OXA and autophagy inhibitor HCQ drugs, which had the ability of targeted inhibition of protective autophagy flux for enhancing chemosensitivity, and simultaneously inhibited cancer distant metastasis. Due to the design, the outer membrane TRAIL-DR4/5 interactions resulted in a tumor targeting drug delivery, leading to the effective tumor biodistribution of OXA and HCQ while sparing on normal cells. The released HCQ could alkalize or damage the lysosome, which blocked the OXA-induced protective autophagy-mediated FAs disassembly, and finally inhibited cancer cell invasion and migration. The in vivo antitumor effect on HCCLM3 subcutaneous and high-metastatic orthotopic tumor models showed TH-NPs treatment can significantly inhibit tumor growth with approximately 75% or 87% decrease. In addition, in the metastatic mouse models, the amount metastatic pulmonary nodules after TH-NPs treatments was largely decreased. Next, we the investigated the detailed mechanism, and found the remarkable antitumor effect of TH-NPs was attributed to the targeting design, of which the enhanced tumor distribution of HCQ overcame OXA-triggered protective autophagy. Then, the degradation of tumor metastatic marker proteins FAs including the E-cadherin and paxillin was reversed. The clinical samples analysis also inspired us to pay much attention to the chemotherapy-induced autophagy and the subsequent metastasis. Collectively, we rationally designed the biomimetic co-delivery nanoparticle TH-NP to efficiently inhibit protective autophagy flux, and then suppress tumors growth and distant metastasis in vitro and vivo. TH-NPs may represent a promising strategy for HCC therapy in clinics.

Additional researches should have been done before this design can be translated. Actually, DR4/5 is also highly expressed in many other types of tumors, such as lung cancer, brain cancer, leukemia and pancreatic cancer, therefore, the drug delivery system could be further exploited as a broad-spectrum anticancer nanoplatform. Moreover, TRAIL–DR4/5 axis was reported to be involved in tumor immune response, and leveraging the functions of TRAIL for enhancing tumor immunotherapy spontaneously would be a promising direction in the delivery system.

Importantly, it is worth mentioning that our delivery system is based on the cell membrane vesicle platform, which involves membrane proteins with high complexity and uncharted risk. However, cells derived from patients may overcome these obstacles and has been used in current medical practice. Overall, the risk of using cell membrane vesicles can be managed. It will be profound to deeply fine-tune our system by developing a targeting tactic that does not rely on the application of TRAIL-DR4/5 axis, such as the use of defined tumor specific molecules for targeting.

## Methods and materials

### Chemicals and regents

Oxaliplatin (OXA), Hydroxychloroquine sulfate (HCQ sulfate) and EDTA were purchased from Medchemexpress. Indocyanine Green-NHS ester (NHS-ICG) was purchased from Ruixi Biological Technology. 1,1'-dioctadecyl-3,3,3',3'-tetramethylindocarbocyanine perchlorate (Dil) and Crystal Violet Staining Solution was obtained from Beyotime. Glutaraldehyde and paraformaldehyde were purchased from Macklin. Phosphatase inhibitor cocktail was bought from Thermo Fisher.

### Cell line and culture

All human HCC cell lines were from the American Type Culture Collection (ATCC). HepG2, Huh-7 and HCCLM3 cells were maintained in DMEM supplemented with 10% fetal bovine serum (FBS), 100 U/mL penicillin, 100U/mL streptomycin. The human umbilical vein endothelial cell (HUVEC) from ATCC was cultured in DMEM supplemented with 10% fetal bovine serum (FBS), 100 U/mL penicillin, 100 U/mL streptomycin.

### Stable expressing cell lines construction

To generate stably overexpressing cell lines, 5 X 10^6^ PFU lentivirus coding TRAIL-GFP, GFP-RFP-LC3, GFP-LC3 or RFP-LC3 were respectively transfected into HUVECs, HEK-293T cells or HCC cell lines. After 48 h transfection, the cells were selected with puromycin to construct stably expressing cell lines.

Then the TRAIL-HUVECs or TRAIL-HEK-293T cells were used as the membrane vesicles derived source cells, and GFP-RFP-LC3, GFP-LC3 or RFP-LC3 HCC cell lines were used for characterization of autophagic flux.

### TRAIL overexpressing cell membrane derivation

The TRAIL overexpressing cell membrane vesicles were harvested according to our previous researches with some modifications [29]. Briefly, the cells were collected and washed with 1X PBS for three times to remove the residual medium. Then, the collected cells were suspended in Tris–HCl buffer (pH = 7.4) contained 10 mM MgCl_2,_ 1X PMSF, 0.2 mM EDTA and phosphatase inhibitor cocktail. Cells were lysed overnight on the shaker at 4 ℃ and then ice bath sonication for about 5 min. The cell lysate solution was centrifuged at 500 g for 10 min at 4 ℃ three times, the resultant supernatant was centrifugated for 30 min at 10,000 g, and then the obtained solution was further centrifugated at 70,000 g for 90 min at 4 ℃. Finally, the acquired pellets were suspended in PBS and then membrane vesicles were quantified by BCA and stored for further applications.

### Preparation and characterization of OXA/HCQ co-delivery NP, H-NPs and TH-NPs

The OXA/HCQ codelivery NPs were synthesized according to the previous method with some modifications. Briefly, 30 mg PLGA (50:50, 0.67 dL/g Lactel Absorbable Polymers) was dissolved with 5 mL DMF (dimethyl formamide) in a round-bottom flask as the oil phase. Then, 5 mL 1% w/v PVA solution containing 20 mg HCQ and 25 mg OXA was used as a water phase, and was added dropwise into the oil phase with stirring overnight. Afterwards, the resultant mixture was transferred to a dialysis bag (MW,10 KD) for 48 h and then lyophilized for the further applications. EE and EC were quantified by the following Eqs. (1) and (2), respectively.

1 $${\text{EE }}\left( \% \right) = {\text{Weight of the drug in NPs}}/{\text{Weight of the drug in feed}} \times {1}00$$

2 $${\text{DLE}}\left( \% \right) = {\text{Weight of the drug in NPs}}/{\text{Weight of the NPs}} \times {1}00$$

H-NPs or TH-NPs were prepared according to the published methods [42]. Briefly, the derived membrane vesicles were mixed with PLGA NPs and then were extruded through the 400 nm and 200 nm Nuclepore membrane (Whatman) under the mini-extruder (Avanti Polar Lipids). The morphology of the NPs, H-NPs and TH-NPs were observed under TEM (JEM-1200, Jeol Ltd, Japan) with uranyl acetate (0.2 wt%) or SEM (Zeiss, GeminiSEM 500). The size and zeta potential were measured on Zetasizer (Malvern, UK).

### In vitro proliferation assay

The three HCC lines including HepG2, Huh-7 and HCCLM3 (500 cells) were seeded into the 6-well plates and then treated with the indicated drugs for 24 h. Then the drugs were removed and the cells were continued to culture until 10 days. The cells were fixed on the ice with 4 ℃ methanol for 10 min and then stained with 0.5% Crystal violet solution (Beyotime Biotechnology) for 20 min. The colonies were counted after washing cells with PBS for three times. Then, 10% acetic acid was added to dissolve the crystal violet and measured its absorbance at 595 nm under Spectrometer (Multiskan GO, Thermo Fisher Scientific) to determine proliferative index.

### In vitro release profile

The release of OXA and HCQ in TH-NPs was determined by dialysis method [46]. Briefly, TH-NPs were put into the dialysis bag (10 KD) and separately put into pH = 7.4 or pH = 5.0 PBS (30 mL) at 37 ℃. The release solution was taken 1 mL at the indicated time point with suppled 1 mL fresh PBS. The drugs content was further analyzed on HPLC.

### Cell immunofluorescence and Western blotting

Cell IF. The cells were cultured in the confocal plates with 60–70% confluence, then the indicated drugs were added and incubated to the time points. After the incubation, the cells were washed three times and then fixed with 4% paraformaldehyde for 10 min. Next, the cells were permeabilized with 0.1% TritonX-100 about 10 min and blocked with 5% goat serum for 1 h at room temperature. Then, the cells were incubated with primary antibodies (1:500) such as cleaved PARP, E-cadherin, paxillin (CST) or Ki67 (Abcam) overnight at 4 ℃. The cells were further washed three times and incubated with FITC-labeled secondary antibody (1:1000, Beyotime) at room temperature for 2 h. After 3 times washing, DAPI was added and stained for about 20 min, and then washed 3 times for the following imaging on Olympus CLSM (FV 1200).

Western blotting. After the treatments of the indicated formulations, the cells were collected and treated with 200 μL RIPA lysis buffer (Beyotime) and 1X PMSF on ice for 30 min. Then the lysate was collected and centrifugated at 12,000 rpm,10 min. 30 μg total protein of the samples was loaded into the SDS-PAGE. After the transferring to PVDF membrane, it was blocked with 5% BSA and incubated with primary antibodies (1:1000) overnight at 4 ℃. The membranes were further washed 4 times every 5 min with TBST, and incubated with HRP-conjugated secondary antibodies (1:5000, Beyotime) for 1 h at room temperature. The signals were detected by Pierce™ ECL (Thermofisher) and the images were observed on Image Lab software. The antibodies used in the research: DR4, DR5, LC3B and p62 from Abcam; E-cadherin and Actin from Bioss and paxillin from CST. Cancer tissues minced and homogenate with RIPA lysis buffer containing PMSF. After the proteins were obtained, the rest of the procedures were similar to that of cells.

### Autophagy analysis

GFP-RFP-LC3 dual reporter assay: the three HCC cell lines stably expressing GFP-RFP-LC3 were seeded in the confocal plates and then treated with the indicated formulations with equivalent dosages of OXA (20 μM) or HCQ (10 μM). The number of GFP or RFP puncta were counted to determine the autophagic flux by using CLSM Olympus CLSM (FV1200).

TEM of autophagic vacuoles assay: the HCC cells were seeded into the 6-well plates and treated with the indicated drugs. The cells were collated into 1.5 mL tubes and fixed in 2.5% glutaraldehyde overnight at 4 ℃. For the caner tissues, it was firstly cut into 1 mm X1mm and then fixed in 2.5% glutaraldehyde overnight at 4 ℃. Then the samples were prepared according to the published protocol [43, 47, 48].

### In vitro invasion and migration assay

For invasion assays, Transwell filter chambers (8 μm pore size, Corning) coated with BD Matrigel were utilized to performed in vitro invasion assay. 5 X 10^4^ HCC cells in 200 μL serum-free DMEM were seeded in the upper chamber of a transwell and 800 μL medium supplemented with 15% FBS was added to the lower chamber. After incubation with the indicated formulations with equivalent dosages of OXA or HCQ, cells migrated through the membrane were fixed with ice-methanol, stained with crystal violet, and counted under optical microscope.

For migration assessment, wound-healing assay was performed. HCC cells were seeded in 6-well plates in advance. The cells were scratched through the cell layer with a 200 μL pipette tip, then cells were incubated with the drugs for 24 h. The relative migration area was counted by Image J.

### Immunofluorescence and IHC staining for tissues

For immunofluorescence staining, the tumors were harvested at the end of the experiment and then were embedded with optimal cutting temperature compound (OCT, Sakura) and stored in − 80 ℃ at once. Then the tissues were performed successive sections of 8 μm under freezing microtome (SLEEMEV). The samples were fixed with 4% paraformaldehyde, permeabilized with 0.1% TritonX-100 and then blocked with 5% goat serum for 1 h at room temperature. Then the samples were incubated with primary antibodies (1:200) including cleaved caspase-3 (CC3), cleaved PARP (C-PARP) and Ki67 overnight at 4 ℃. After three times washing, fluorescence-labeled secondary antibodies (1:1000) were incubated with the samples for 1 h at room temperature. Finally, the slides were observed under Olympus CLSM (FV1200). For IHC staining, the tissues were embedded in paraffin and sectioned into 5 μm slices. Then the samples were deparaffinized and rehydrated in a graded ethanol series. Next, the sections were incubated in 0.3% hydrogen peroxide for 15 min to block the activity of endogenous peroxidase and then performed antigen retrieval in citrate buffer (10 mM, pH = 6) for 30 min. After three times washing, the samples were incubated in blocking buffer (5% goat serum) for 1 h at room temperature.

### Antitumor activity in subcutaneous HCCLM3 tumor-bearing mice

When the tumor volume of HCCLM3 tumor-bearing mice reached about 150 mm^3^, the mice were randomly into seven groups (n = 8). Mice received i.v. injection of (1) Saline, (2) HCQ, (3) OXA, (4) HCQ + OXA, (5) NPs, (6) H-NPs, (7) TH-NPs, with an equivalent dosage of OXA (10 mg/kg) or HCQ (20 mg/kg). The therapeutic procedure was shown in Fig. 4A. On 18th day of the treatment, three mice were used to detect the drugs content in vivo, while the other mice were used to verify the anticancer effect. Tumor volumes were measured every three days and the average tumor volume was calculated by using the formula: V(volume) = (length X width^2^)/2. On the 30th day, the mice were sacrificed and the tumor tissues were collected for the further IHC analysis.

### Antitumor activity in orthotopic HCCLM3 tumor models

To further examine the antitumor and antimetastatic efficacy of TH-NPs in vivo, the orthotopic liver models were developed. Briefly, 6-week male nude mice were anesthetized with isoflurane, and then 1 X 10^6^ HCCLM3-Luc cells suspended in 40 μL DMEM/BD Matrigel were inoculated in the left hepatic lobe by using a microsyringe. The following therapeutic regimen was shown in Fig. 5A. Seven days after the inoculation, mice received i.v. injection of Saline, NPs, H-NPs, and TH-NPs, with equivalent dose of OXA (10 mg/kg) or HCQ (20 mg/kg). All of mice were sacrificed 7 weeks later, and the tumor or lung was collected for further autophagic analysis or H&E staining.

### Antimetastatic activity in HCC lung metastasis tumor models

For the lung metastasis model, 5 X 10^5^ HCC cells suspended in 200 μL Hank’s solution were injected into the lateral tail vein of nude mice. After 48 h, the mice were injected with saline or TH-NPs (OXA: 10 mg/kg; HCQ: 20 mg/kg) every three days for seven times. For investigation of the antimetastatic ability of TH-NPs, the mice were sacrificed after 6 weeks and the lungs were collected for the further H&E staining for analyzing the tumor nodules on the lungs.

### Statistics

All data were shown as Mean ± SD with at least three times repeat. Student’s *t* test was used for two-group comparisons, and one-way analysis variance (ANOVA) with Tukey’s correction for multiple comparations was used for comparing three or more groups. Statistical significance shown in the research was P < 0.05, **P < 0.01, ***P < 0 0.001.

## Supplementary Information

**Additional file 1: Figure S1.** Autophagy examination of HCC cells. **Figure S2**. Verification of TRAIL expression and death receptors. **Figure S3**. Characterization of nanoparticles. **Figure S4.** Tumor targeting of TH-NP.  **Figure S5.** Autophagy analysis of HCC cells. **Figure S6.** TH-NPs enhance chemotherapeutic efficacy of OXA through inhibiting autophagy. **Figure S7.** Effect of autophagy inhibition on migration and invasion of HCC cells. **Figure S8.** Effect of autophagy inhibition on antitumor efficacy in vivo. **Figure S9.** Effect of autophagy inhibition on antimetastatic efficacy in vivo. Figure S10. Blockade of autophagy leads to the reverse of FAs turnover.

## Data Availability

Most of the datasets supporting the conclusions of this article are included within this article. The datasets used or analyzed during the current study are available on reasonable request.

## References

[1] Palmieri LJ, Dermine S, Coriat R (2019). Potential areas of interest in a trial of sorafenib plus hepatic arterial infusion of oxaliplatin, fluorouracil, and leucovorin for hepatocellular carcinoma. JAMA Oncol.

[2] Ding ZB, Hui B, Shi YH, Zhou J, Peng YF, Gu CY, Yang H (2011). Autophagy activation in hepatocellular carcinoma contributes to the tolerance of oxaliplatin via reactive oxygen species modulation. Clin Cancer Res.

[3] Cai Q, Wang S, Jin L, Weng M, Zhou D, Wang J, Tang Z (2019). Long non-coding RNA GBCDRlnc1 induces chemoresistance of gallbladder cancer cells by activating autophagy. Mol Cancer.

[4] Amaravadi RK, Kimmelman AC, Debnath J (2019). Targeting autophagy in cancer: recent advances and future directions. Cancer Discov.

[5] Ou J, Peng Y, Yang W, Zhang Y, Hao J, Li F, Chen Y (2019). ABHD5 blunts the sensitivity of colorectal cancer to fluorouracil via promoting autophagic uracil yield. Nat Commun.

[6] Chang YS, Jalgaonkar SP, Middleton JD, Hai T (2017). Stress-inducible gene Atf3 in the noncancer host cells contributes to chemotherapy-exacerbated breast cancer metastasis. Proc Natl Acad Sci U S A.

[7] Keklikoglou I, Cianciaruso C, Güç E, Squadrito ML, Spring LM, Tazzyman S, Lambein L (2019). Chemotherapy elicits pro-metastatic extracellular vesicles in breast cancer models. Nat Cell Biol.

[8] Fong MY, Zhou W, Liu L, Alontaga AY, Chandra M, Ashby J, Chow A (2015). Breast-cancer-secreted miR-122 reprograms glucose metabolism in premetastatic niche to promote metastasis. Nat Cell Biol.

[9] Bryant KL, Stalnecker CA, Zeitouni D, Klomp JE, Peng S, Tikunov AP, Gunda V (2019). Combination of ERK and autophagy inhibition as a treatment approach for pancreatic cancer. Nat Med.

[10] Kinsey CG, Camolotto SA, Boespflug AM, Guillen KP, Foth M, Truong A, Schuman SS (2019). Protective autophagy elicited by RAF–>MEK–>ERK inhibition suggests a treatment strategy for RAS-driven cancers. Nat Med.

[11] Chen M, Yang D, Sun Y, Liu T, Wang W, Fu J, Wang Q (2021). In situ self-assembly nanomicelle microneedles for enhanced photoimmunotherapy via autophagy regulation strategy. ACS Nano.

[12] Venturelli S, Berger A, Weiland T, Zimmermann M, Häcker S, Peter C, Wesselborg S (2011). Dual antitumour effect of 5-azacytidine by inducing a breakdown of resistance-mediating factors and epigenetic modulation. Gut.

[13] Ruiz de Galarreta M, Bresnahan E, Molina-Sánchez P, Lindblad KE, Maier B, Sia D, Puigvehi M (2019). β-catenin activation promotes immune escape and resistance to anti-PD-1 therapy in hepatocellular carcinoma. Cancer Discov.

[14] Gump JM, Staskiewicz L, Morgan MJ, Bamberg A, Riches DWH, Thorburn A (2014). Autophagy variation within a cell population determines cell fate through selective degradation of Fap-1. Nat Cell Biol.

[15] Huang X, Gan G, Wang X, Xu T, Xie W (2019). The HGF-MET axis coordinates liver cancer metabolism and autophagy for chemotherapeutic resistance. Autophagy.

[16] Xu WP, Liu JP, Feng JF, Zhu CP, Yang Y, Zhou WP, Ding J (2020). miR-541 potentiates the response of human hepatocellular carcinoma to sorafenib treatment by inhibiting autophagy. Gut.

[17] Peng YF, Shi YH, Ding ZB, Ke AW, Gu CY, Hui B, Zhou J (2013). Autophagy inhibition suppresses pulmonary metastasis of HCC in mice via impairing anoikis resistance and colonization of HCC cells. Autophagy.

[18] Liu C, Sun L, Yang J, Liu T, Yang Y, Kim SM, Ou X (2018). FSIP1 regulates autophagy in breast cancer. Proc Natl Acad Sci U S A.

[19] Wang Y, Yin S, Zhang L, Shi K, Tang J, Zhang Z, He Q (2018). A tumor-activatable particle with antimetastatic potential in breast cancer via inhibiting the autophagy-dependent disassembly of focal adhesion. Biomaterials.

[20] Sharifi MN, Mowers EE, Drake LE, Collier C, Chen H, Zamora M, Mui S (2016). Autophagy promotes focal adhesion disassembly and cell motility of metastatic tumor cells through the direct interaction of paxillin with LC3. Cell Rep.

[21] Liu H, Ma Y, He HW, Zhao WL, Shao RG (2017). SPHK1 (sphingosine kinase 1) induces epithelial-mesenchymal transition by promoting the autophagy-linked lysosomal degradation of CDH1/E-cadherin in hepatoma cells. Autophagy.

[22] Ma H, Li Y, Wang X, Wu H, Qi G, Li R, Yang N (2019). PBK, targeted by EVI1, promotes metastasis and confers cisplatin resistance through inducing autophagy in high-grade serous ovarian carcinoma. Cell Death Dis.

[23] Karagiannis GS, Pastoriza JM, Wang Y, Harney AS, Entenberg D, Pignatelli J, Sharma VP (2017). Neoadjuvant chemotherapy induces breast cancer metastasis through a TMEM-mediated mechanism. Sci Transl Med.

[24] Zhang F, Wang H, Yu J, Yao X, Yang S, Li W, Xu L (2021). LncRNA CRNDE attenuates chemoresistance in gastric cancer via SRSF6-regulated alternative splicing of PICALM. Mol Cancer.

[25] Hu F, Song D, Yan Y, Huang C, Shen C, Lan J, Chen Y (2021). IL-6 regulates autophagy and chemotherapy resistance by promoting BECN1 phosphorylation. Nat Commun.

[26] Xin X, Du X, Xiao Q, Azevedo HS, He W, Yin L (2019). Drug nanorod-mediated intracellular delivery of microRNA-101 for self-sensitization via autophagy inhibition. Nanomicro Lett.

[27] Xu F, Li X, Huang X, Pan J, Wang Y, Zhou S (2020). Development of a pH-responsive polymersome inducing endoplasmic reticulum stress and autophagy blockade. Sci Adv.

[28] Mauthe M, Orhon I, Rocchi C, Zhou X, Luhr M, Hijlkema KJ, Coppes RP (2018). Chloroquine inhibits autophagic flux by decreasing autophagosome-lysosome fusion. Autophagy.

[29] Ji Y, Liu X, Li J, Xie X, Huang M, Jiang J, Liao YP (2020). Use of ratiometrically designed nanocarrier targeting CDK4/6 and autophagy pathways for effective pancreatic cancer treatment. Nat Commun.

[30] Ruan S, Xie R, Qin L, Yu M, Xiao W, Hu C, Yu W (2019). Aggregable nanoparticles-enabled chemotherapy and autophagy inhibition combined with Anti-PD-L1 antibody for improved glioma treatment. Nano Lett.

[31] Levy JMM, Towers CG, Thorburn A (2017). Targeting autophagy in cancer. Nat Rev Cancer.

[32] Padmanaban V, Krol I, Suhail Y, Szczerba BM, Aceto N, Bader JS, Ewald AJ (2019). E-cadherin is required for metastasis in multiple models of breast cancer. Nature.

[33] Nguyen-Ngoc KV, Cheung KJ, Brenot A, Shamir ER, Gray RS, Hines WC, Yaswen P (2012). ECM microenvironment regulates collective migration and local dissemination in normal and malignant mammary epithelium. Proc Natl Acad Sci U S A.

[34] Zhou W, Gong L, Wu Q, Xing C, Wei B, Chen T, Zhou Y (2018). PHF8 upregulation contributes to autophagic degradation of E-cadherin, epithelial-mesenchymal transition and metastasis in hepatocellular carcinoma. J Exp Clin Cancer Res.

[35] He M, Li Q, Zou R, Shen J, Fang W, Tan G, Zhou Y (2019). Sorafenib plus hepatic arterial infusion of oxaliplatin, fluorouracil, and leucovorin vs sorafenib alone for hepatocellular carcinoma with portal vein invasion: a randomized clinical trial. JAMA Oncol.

[36] Selvakumaran M, Amaravadi RK, Vasilevskaya IA, O'Dwyer PJ (2013). Autophagy inhibition sensitizes colon cancer cells to antiangiogenic and cytotoxic therapy. Clin Cancer Res.

[37] Wang X, Li M, Ren K, Xia C, Li J, Yu Q, Qiu Y (2020). On-demand autophagy cascade amplification nanoparticles precisely enhanced oxaliplatin-induced cancer immunotherapy. Adv Mater.

[38] Yong T, Zhang X, Bie N, Zhang H, Zhang X, Li F, Hakeem A (2019). Tumor exosome-based nanoparticles are efficient drug carriers for chemotherapy. Nat Commun.

[39] Deng G, Sun Z, Li S, Peng X, Li W, Zhou L, Ma Y, Gong P, Cai L (2018). Cell-membrane immunotherapy based on natural killer cell membrane coated nanoparticles for the effective inhibition of primary and abscopal tumor growth. ACS Nano.

[40] Liu X, Liu C, Zheng Z, Chen S, Pang X, Xiang X, Tang J (2019). Vesicular antibodies: a bioactive multifunctional combination platform for targeted therapeutic delivery and cancer immunotherapy. Adv Mater.

[41] Zhang P, Zhang L, Qin Z, Hua S, Guo Z, Chu C, Lin H (2018). Genetically engineered liposome-like nanovesicles as active targeted transport platform. Adv Mater.

[42] Shi Y, Xie F, Rao P, Qian H, Chen R, Chen H, Li D (2020). TRAIL-expressing cell membrane nanovesicles as an anti-inflammatory platform for rheumatoid arthritis therapy. J Control Release.

[43] Shi Y, Wang J, Liu J, Lin G, Xie F, Pang X, Pei Y (2020). Oxidative stress-driven DR5 upregulation restores TRAIL/Apo2L sensitivity induced by iron oxide nanoparticles in colorectal cancer. Biomaterials.

[44] Shi Y, Pang X, Wang J, Liu G (2018). NanoTRAIL-oncology: a strategic approach in cancer research and therapy. Adv Healthc Mater.

[45] Liu X, Yuan L, Zhang L, Mu Y, Li X, Liu C, Lv P (2018). Bioinspired artificial nanodecoys for hepatitis B virus. Angew Chem Int Ed Engl.

[46] Zhang P, Wang J, Chen H, Zhao L, Chen B, Chu C, Liu H (2018). Tumor microenvironment-responsive ultrasmall nanodrug generators with enhanced tumor delivery and penetration. J Am Chem Soc.

[47] Yang Z, Gao D, Guo X, Jin L, Zheng J, Wang Y, Chen S (2020). Fighting immune cold and reprogramming immunosuppressive tumor microenvironment with red blood cell membrane-camouflaged nanobullets. ACS Nano.

[48] Chen Y, Chen Y, Zhang J, Cao P, Su W, Deng Y, Zhan N (2020). Fusobacterium nucleatum promotes metastasis in colorectal cancer by activating autophagy signaling via the upregulation of CARD3 expression. Theranostics.

